# Comprehensive Functional Analysis of *Escherichia coli* Ribosomal RNA Methyltransferases

**DOI:** 10.3389/fgene.2020.00097

**Published:** 2020-02-27

**Authors:** Philipp Pletnev, Ekaterina Guseva, Anna Zanina, Sergey Evfratov, Margarita Dzama, Vsevolod Treshin, Alexandra Pogorel’skaya, Ilya Osterman, Anna Golovina, Maria Rubtsova, Marina Serebryakova, Olga V. Pobeguts, Vadim M. Govorun, Alexey A. Bogdanov, Olga A. Dontsova, Petr V. Sergiev

**Affiliations:** ^1^ Department of Chemistry, Lomonosov Moscow State University, Moscow, Russia; ^2^ Center of Life Sciences, Skolkovo Institute of Science and Technology, Moscow, Russia; ^3^ Shemyakin-Ovchinnikov Institute of Bioorganic Chemistry, Moscow, Russia; ^4^ Faculty of Bioengineering and Bioinformatics, Lomonosov Moscow State University, Moscow, Russia; ^5^ Belozersky Institute of Physico-Chemical Biololgy, Lomonosov Moscow State University, Moscow, Russia; ^6^ Federal Research and Clinical Centre of Physical-Chemical Medicine, Moscow, Russia; ^7^ Institute of Functional Genomics, Lomonosov Moscow State University, Moscow, Russia

**Keywords:** ribosome, rRNA, methyltransferase, modification, *Escherichia coli*

## Abstract

Ribosomal RNAs in all organisms are methylated. The functional role of the majority of modified nucleotides is unknown. We systematically questioned the influence of rRNA methylation in *Escherichia coli* on a number of characteristics of bacterial cells with the help of a set of rRNA methyltransferase (MT) gene knockout strains from the Keio collection. Analysis of ribosomal subunits sedimentation profiles of the knockout strains revealed a surprisingly small number of rRNA MT that significantly affected ribosome assembly. Accumulation of the assembly intermediates was observed only for the *rlmE* knockout strain whose growth was retarded most significantly among other rRNA MT knockout strains. Accumulation of the 17S rRNA precursor was observed for *rsmA*(*ksgA*) knockout cells as well as for cells devoid of functional *rsmB* and *rlmC* genes. Significant differences were found among the WT and the majority of rRNA MT knockout strains in their ability to sustain exogenous protein overexpression. While the majority of the rRNA MT knockout strains supported suboptimal reporter gene expression, the strain devoid of the *rsmF* gene demonstrated a moderate increase in the yield of ectopic gene expression. Comparative 2D protein gel analysis of rRNA MT knockout strains revealed only minor perturbations of the proteome.

## Introduction

Ribosomal RNA (rRNA) of all organisms contains modified nucleosides (Sergiev et al., 2011). The functional role of such modifications is unknown in many cases. Some modifications were shown to be required for proper ribosome assembly (Connolly et al., 2008), interaction with ribosomal ligands (Burakovsky et al., 2012), antibiotic resistance (Weisblum, 1995), and the correct functioning of particular regulatory mechanisms (Vazquez-Laslop et al., 2008; Vazquez-Laslop et al., 2010; Prokhorova et al., 2013). In 2012, the entire list of *E. coli* rRNA methyltransferase genes was completed (Golovina et al., 2012). Not a single modification of rRNA was found to be essential for bacterial cell survival, although earlier studies indicated that the lack of several modifications might cumulatively have a deleterious effect on ribosome activity (Green and Noller, 1996). Obviously, none of the rRNA modifications is required for the general ability of a ribosome to synthesize proteins. However, in a living cell, a ribosome should synthesize the proteins quickly, at the right proportions, and in a cost-effective manner (Li et al., 2014). Ribosomal RNA modification might contribute to the fine-tuning of particular gene expression mechanisms (Sergiev et al., 2012; Prokhorova et al., 2013) or contribute to the efficiency of protein biosynthesis in general. At favorable growth conditions, the inefficiency of protein biosynthesis might be tolerated, while an increased load on the protein biosynthesis machinery, such as in the artificial case of foreign gene overexpression, or when it is necessary to replace damaged proteins, modification of rRNA might play a role. In this work we systematically studied the influence of rRNA methyltransferase gene knockouts (**Table 1**) on bacterial growth, the accumulation of assembly intermediates, deviations in the proteome, and the ability to sustain excessive protein synthesis.

**Table 1:** List of *E. coli* rRNA MT coding genes and the phenotypes of their knockouts.

Nucleotide	Enzyme	Reference	Growth of knockout strain/growth at overexpression*	CER expression**	FastFT expression***	Accumulation of assembly intermediates and 17S rRNA precursor	Expression level**** and timing
16S rRNA							
527 m^7^G	RsmG	Okamoto et al., 2007	+++\++	+++	+++	−/−	+++, 3h
966 m^2^G	RsmD	Lesnyak et al., 2007	+++\+++	+	+++	−/−	+++, 2h
967 m^5^C	RsmB	Tscherne et al., 1999a; Gu et al., 1999	+++\+++	++	++	−/++	++++, 4h
1207 m^2^G	RsmC	Tscherne et al., 1999b	+++\+++	++	+++	−/−	++++, 2h
1402 m^4^Cm	RsmI, RsmH	Kimura and Suzuki, 2010	+++\+	+	+/+++^#^	−/−	+, 2h
1407 m^5^C	RsmF	Andersen and Douthwaite, 2006	+++\+++	++++	++++	−/−	+++, 2h
1498 m^3^U	RsmE	Basturea et al., 2006	+++\+++	+++	+++	−/−	+++, 2h
1516 m^2^G	RsmJ	Basturea et al., 2012	+++\+++	+	++	−/−	+++, 2h
1518/9 m^6^_2_A	RsmA	Helser et al., 1972; Poldermans et al., 1979	+++\+++	+	+	−/++	++++, 2h
23S rRNA							
745 m^1^G	RlmA	Gustafsson and Persson, 1998	+++\++	+	+/+++^#^	−/−.	++++, 2h
747 m^5^U	RlmC	Madsen et al., 2003	+++\++	+	+++	−/++	++, 2h
1618 m^6^A	RlmF	Sergiev et al., 2008	+++\++	+	+++	−/−	+++, 1–7h
1835 m^2^G	RlmG	Sergiev et al., 2006	+++\+++	++	++	−/−	+++, 2h
1915 m^3^ Ψ	RlmH	Purta et al., 2008a; Ero et al., 2008	+++\+++	+	++	−/−	+++, 2h
1939 m^5^U	RlmD	Agarwalla et al., 2002; Madsen et al., 2003	+++\+++	+++	+++	−/−	+++, 2h
1962 m^5^C	RlmI	Purta et al., 2008b	+++\+	+	++	−/−	+++, 2h
2030 m^6^A	RlmJ	Golovina et al., 2012	+++\+++	+	+++	−/−	++++, 2h
2069 m^7^G, 2445 m^2^G	RlmKL	Kimura et al., 2012	+++\+++	+	+++	−/−	+++, 2h
2251 Gm	RlmB	Lovgren and Wikstrom, 2001	+++\+++	++	+++	−/−	+++, 2h
2498 Cm	RlmM	Purta et al., 2009	+++\+++	+	++	+?/−	+++, 2h
2503 m^2^A	RlmN	Toh and Mankin, 2008	+++\++	+	+++	+?/-	+++, 2h
2552 Um	RlmE	Caldas et al., 2000; Bugl et al., 2000	+\+	+	+/++^#^	+++/−	++++, 3h
	WT		+++	+++	+++	−/−	

*+++ corresponds to doubling times 40–60 min, ++ 60–70 min, + > 70 min. ** CER fluorescence after overnight growth at inducing conditions ++++ > 6·10^5^, +++ 4−6·10^5^, ++ 2−4·10^5^, + < 4·10^5^. ***Fluorescence intensity of the blue, newly produced form of the FastFT at the exponential growth conditions ++++ > 2·10^3^, +++1–2·10^3^, ++5·10^2^–10^3^, + < 5·10^2^. ****Expression levels at a timepoint with maximal expression. ++++ > 10^−4^, +++ > 10^−5^, ++ > 10^−6^, + < 10^−6^ relative to the 16S rRNA. ^#^During growth of these strains transformed by FastFT expression plasmid a significant amount of non-fluorescent cells were accumulated.

## Results

### Expression of rRNA Methyltransferase Genes at Different Stages of Bacterial Culture Growth

**Figure 1:**
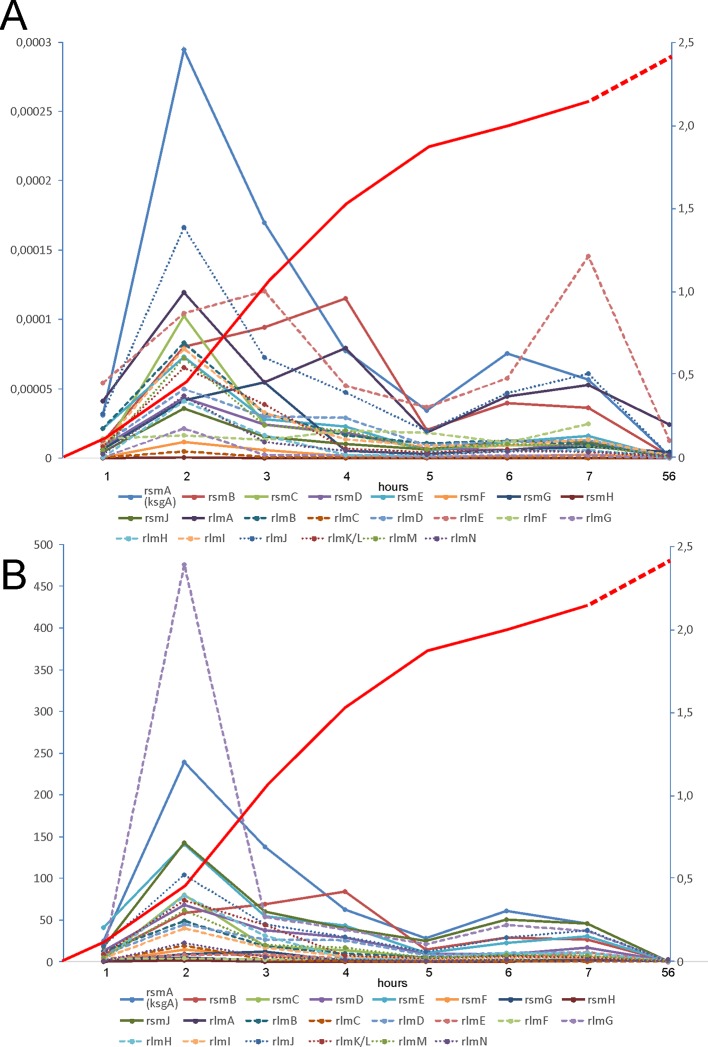
Expression of rRNA methyltransferase genes at different phases of *E. coli* culture growth. **(A)** Amounts of rRNA methyltransferases mRNA relative to the amount of 16S rRNA as revealed by RT qPCR (left scale). **(B)** Change in the proportion of rRNA methyltransferases mRNA to the 16S rRNA relative to that in the stationary phase (left scale). The keys to the graphs are shown below the panels. Red curves (right scale) correspond to the A_600_ of the cell culture.

Gene expression analysis in specific conditions might give a hint about its specialized function. Various genes, coding for rRNA modification enzymes, show distinct expression patterns according to the databases on a global analysis of gene expression in a variety of conditions (Sergiev et al., 2012). We monitored expression of rRNA methyltransferase genes experimentally at different stages of bacterial culture growth using RT-qPCR (**Figure 1**). In the computer analysis of GEO profiles of gene expression focused on rRNA modification genes (Sergiev et al., 2012) we noticed that the majority of rRNA methylation genes, except for *rsmA*(*ksgA*) and *rlmE*, are coexpressed with other genes associated with fast growth. Here, we demonstrated experimentally that the expression of the majority of rRNA methyltransferase genes is induced at an early exponential phase (**Figure 1A**). The amount of mRNA molecules coding for different rRNA methyltransferases normalized to the amount of rRNA differs by three orders of magnitude. We observed the highest expression level for the *rsmA*(*ksgA*) gene and the lowest for *rsmH*. Analysis of the growth phase dependencies of expression (**Figure 1B**) revealed a group of rRNA methyltransferase genes, namely, *rlmN*, *rlmC*, *rsmF*, *rsmG*, *rlmA*, *rlmF*, and *rsmH*, with nearly constitutive expression. The remaining rRNA MT coding genes demonstrated a variable extent of induction from small to moderate for *rsmC*, *rlmH*, *rlmKL*, *rsmD*, *rlmM*, *rlmB*, *rlmD*, *rlmI*, high for *rsmE*, *rsmJ*, *rlmJ*, and extreme for *rlmG* and *rsmA*(*ksgA*). Genes coding for *rsmB* and *rlmE* have specific expression patterns (**Figure 1**). The *RsmB* gene is transcribed at a later growth stage, the maximal expression level being attained at 4 h after dilution of the culture in fresh media. The gene coding for *RlmE* is transcribed almost constitutively with the maximal level of expression reached after 7 h of bacterial culture dilution in fresh media.

### Influence of rRNA Methyltransferase Genes Inactivation on the Accumulation of Ribosome Assembly Intermediates

**Figure 2:**
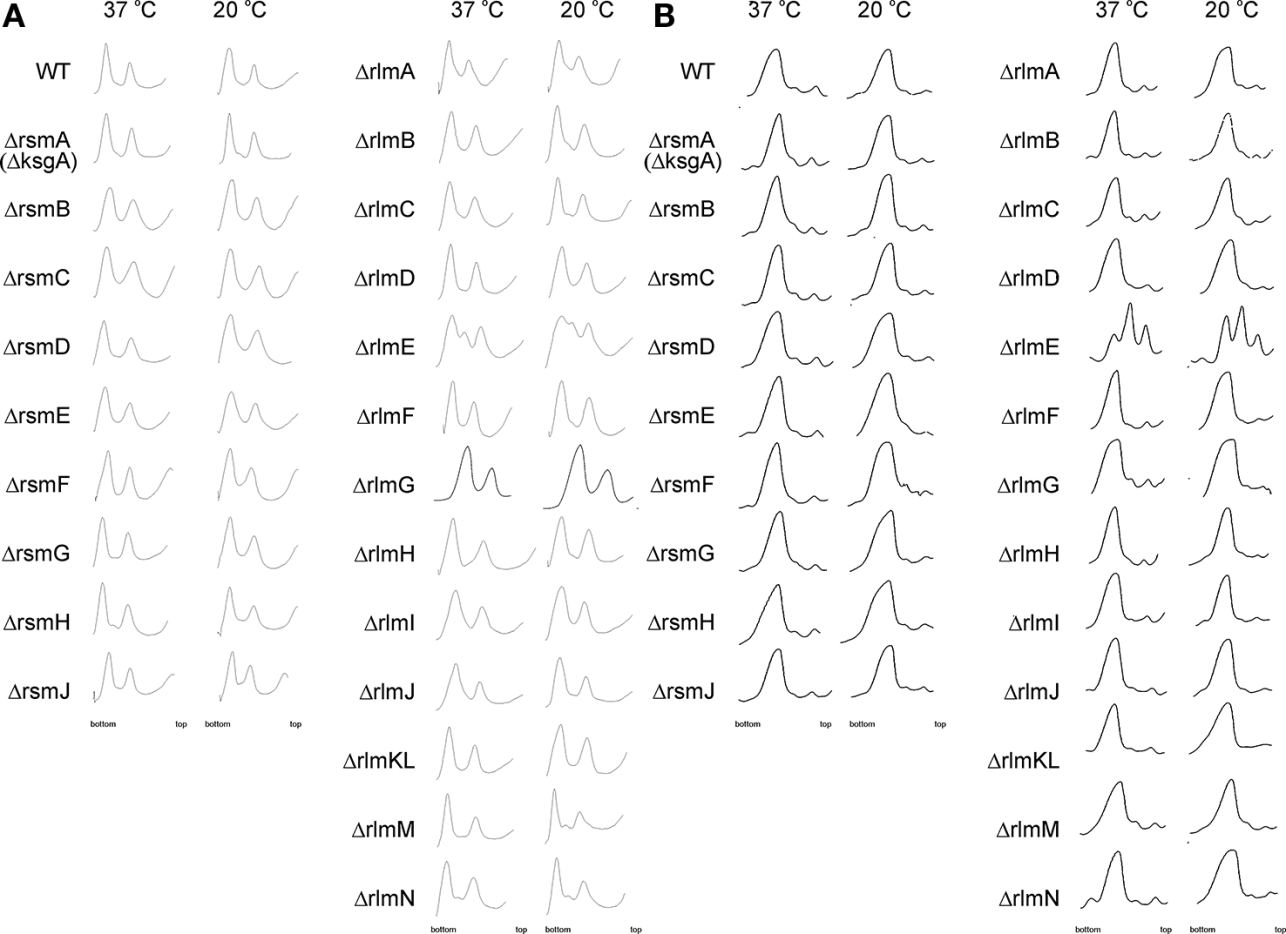
Accumulation of ribosomal subunits assembly intermediates in the *E. coli* strains with inactivation of the 16S rRNA and 23S rRNA methyltransferase genes marked on the left side. WT corresponds to the parental isogeneic strain carrying all set of rRNA methyltransferase genes. Shown are sucrose gradient centrifugation profiles at subunit dissociation conditions (1 mM magnesium ions concentration) **(A)** and association conditions (10 mM magnesium ions concentration) **(B)**. Left panels correspond to the cells grown at 37°C, while that to the right correspond to the cells grown at 20°C known to exacerbate ribosomal subunits assembly defects.

A function of rRNA modification enzymes in ribosome assembly was proposed for RlmE and RsmA(KsgA) rRNA methyltransferases. The knockout of the former caused an accumulation of assembly intermediates and slow growth (Bugl et al., 2000; Caldas et al., 2000; Hager et al., 2002; Arai et al., 2015), which could be suppressed by overexpression of small GTPases Obg and EngA (Tan et al., 2002). RsmA(KsgA) was proposed to be the ribosome biogenesis factor, utilizing its methyltransferase activity to trigger its dissociation upon successful completion of the 30S subunit assembly (Connolly et al., 2008). Involvement of other rRNA methyltransferases in the ribosome assembly pathway could be hypothesized (Sergiev et al., 2011). We used a collection of rRNA methyltransferase knockout strains (Baba et al., 2006) to systematically study the accumulation of assembly intermediates of ribosomal subunits. Knockout strains were grown in a rich medium at 37°C and 20°C. Low temperature slows conformational rearrangements of RNA and is known to aggravate ribosome assembly defects (Shajani et al., 2011); thus, at 20°C we expected to reveal more severe defects then at 37°C. Sucrose density centrifugations of the rRNA methyltransferase gene knockout cell lysates were done at low, 1 mM (**Figure 2A**) and high 10 mM (**Figure 2B**) magnesium ion concentrations to reveal any differences in accumulation of assembly intermediates or subunit association. Only the *rlmE* knockout strain previously known to accumulate a significant amount of assembly intermediates (Bugl et al., 2000; Caldas et al., 2000; Hager et al., 2002; Arai et al., 2015) demonstrated such an effect at both magnesium ion concentrations. In addition, small peaks of presumably assembly intermediates might be observed on the sucrose gradients at a low magnesium ion concentration of the extracts from Δ*rlmM* and Δ*rlmN* strains.

Surprisingly, none of the other rRNA methyltransferase knockout strains demonstrated the accumulation of ribosomal subunits assembly intermediates with sedimentation properties different from that of mature ribosomal subunits, arguing against the essentiality of the corresponding rRNA modifications for ribosome assembly.

**Figure 3:**
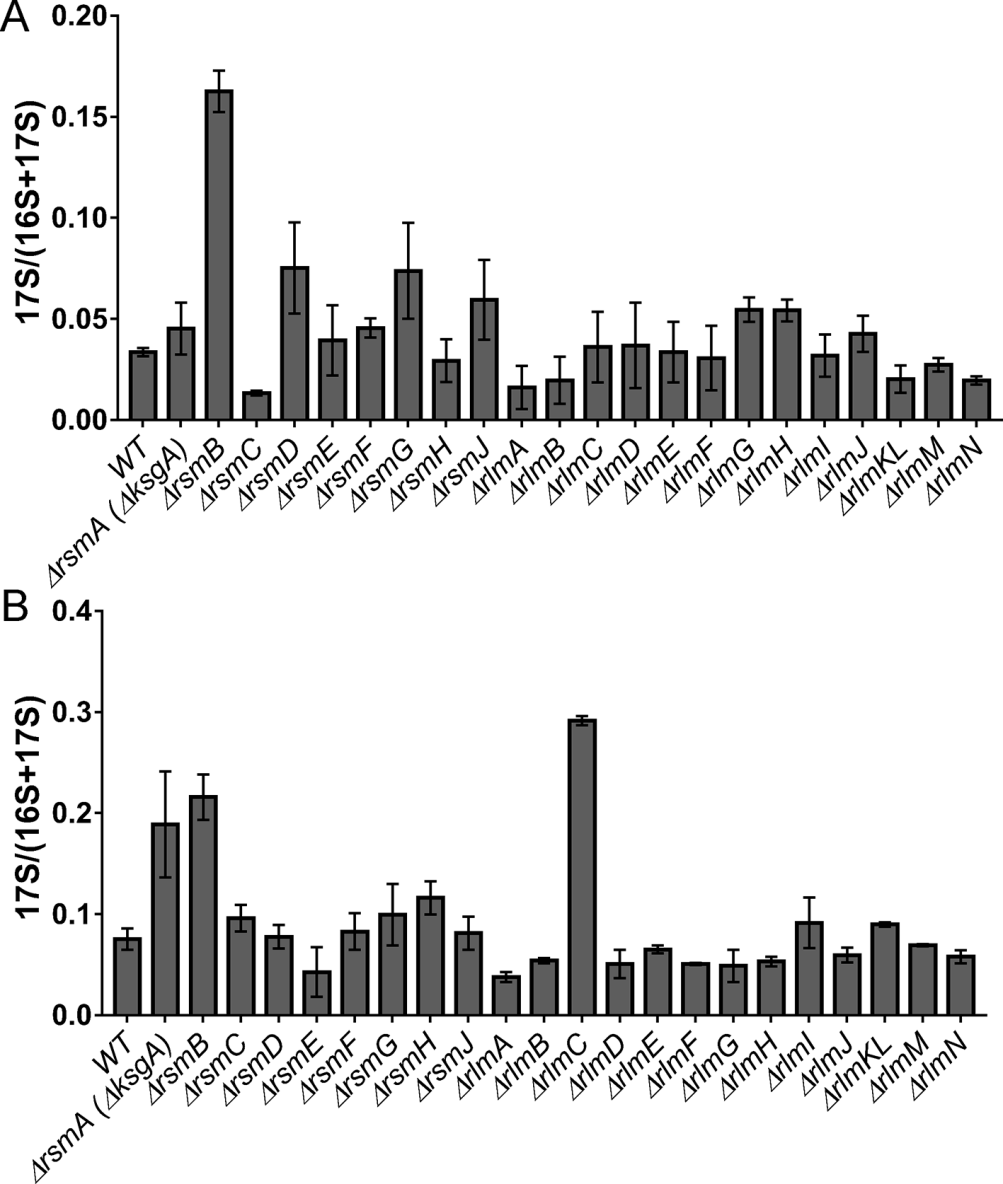
Accumulation of 17S rRNA precursor in the *E. coli* strains with inactivation of the 16S rRNA and 23S rRNA methyltransferase genes. Shown is the 17S rRNA to 16S+17S rRNA ratio determined by RT qPCR. Knockout strains are marked below the bars. (Panel **A**) corresponds to the cells grown at the 37°C, while (panel **B**) to the cells grown at the 20°C.

Nucleolytic processing of the 17S rRNA precursor resulting in the formation of the 16S rRNA happens in the late stage of small ribosomal subunit assembly (Smith et al., 2018). For the few strains deficient in rRNA modification enzymes (Gutgsell et al., 2005; Connolly et al., 2008) excessive accumulation of the 17S rRNA precursor was demonstrated. However, up to now, no systematic study of rRNA modification’s influence on the processing of the 17S rRNA precursor was available. We decided to analyze 17S rRNA to 16S rRNA ratio in the cells devoid of each of the rRNA methyltransferases (**Figure 3**) with the help of the RT qPCR. An accumulation of the 17S rRNA precursor was detected for cells of the Δ*rsmA*(Δ*ksgA*) strain grown at a low temperature, in agreement with previously published data (Connolly et al., 2008). In addition, a substantial increase in the amount of the 17S rRNA processing intermediate was detected for the cells of the Δ*rsmB* and Δ*rlmC* strains. For the former, a higher concentration of the precursor was detected at both tested temperatures, while for the latter, 17S was accumulated at 20°C.

### Influence of rRNA Methyltransferase Gene Inactivation on the Growth Rate of Bacteria

**Figure 4:**
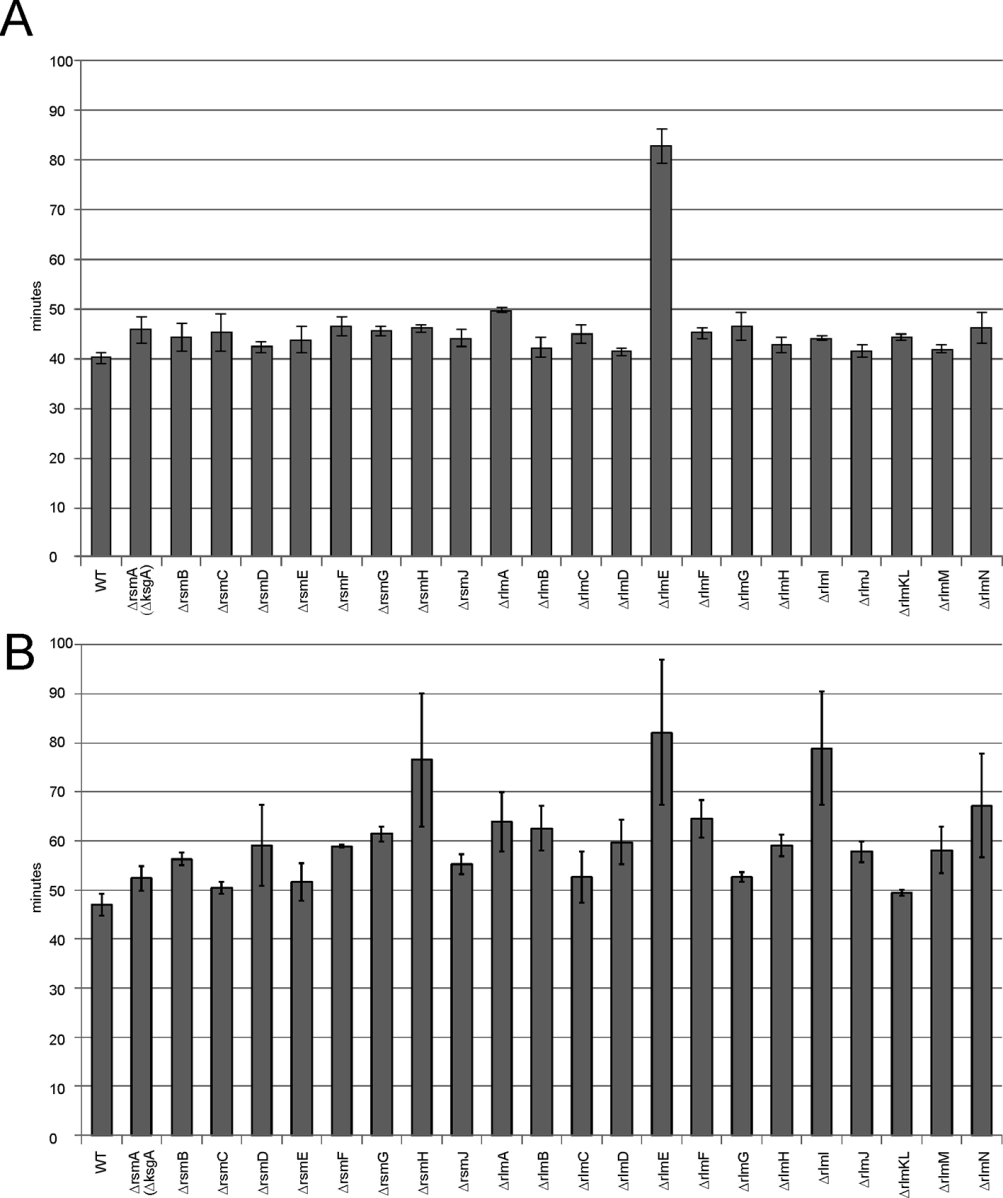
Influence of rRNA methyltransferase gene inactivation on the growth rates of bacteria. **(A)** Doubling times of the strains devoid of the rRNA methyltransferase genes. Inactivated genes are labeled below the graph. WT corresponds to the parental isogeneic strain carrying a whole set of rRNA methyltransferase genes. **(B)** Doubling times of the same strains carrying a plasmid coding for the cerulean fluorescent protein (CER) and red fluorescent protein (RFP) reporter proteins at induction of reporter gene expression.

Protein biosynthesis requires a large share of cellular resources. Any deviation from optimal protein synthesis efficiency should influence the doubling time of bacteria. We consistently measured the growth rates of bacteria lacking rRNA methyltransferase genes (**Figure 4**). At optimal conditions in a rich medium (**Figure 4A**) only the Δ*rlmE* strain demonstrated a significant, nearly twofold increase in the doubling time. Such a result correlates well with the accumulation of assembly intermediates in this strain.

Overexpression of an exogenous protein leads to the unproductive waste of biosynthetic protein resources. It may lead to significant growth retardation if the protein biosynthesis machinery would operate at suboptimal efficiency. To evaluate the influence of exogenous gene expression on cell growth, we used a reporter plasmid carrying the red fluorescent protein (*rfp*) gene under a constitutive T5 promoter and the cerulean fluorescent protein (*cer*) gene under the inducible Tet promoter. Growth rates were measured for rRNA methyltransferase knockout strains upon overexpression of the CER fluorescent protein gene (**Figure 4B**). More knockout strains revealed the difference from the wild-type parental strain in doubling time; in addition to the strain lacking RlmE methyltransferase, significant growth retardation was found in the strains lacking RsmH and RlmI rRNA MTs.

### Influence of rRNA Methyltransferase Gene Inactivation on the Efficiency of Constitutive and Induced Exogenous Protein Synthesis

**Figure 5:**
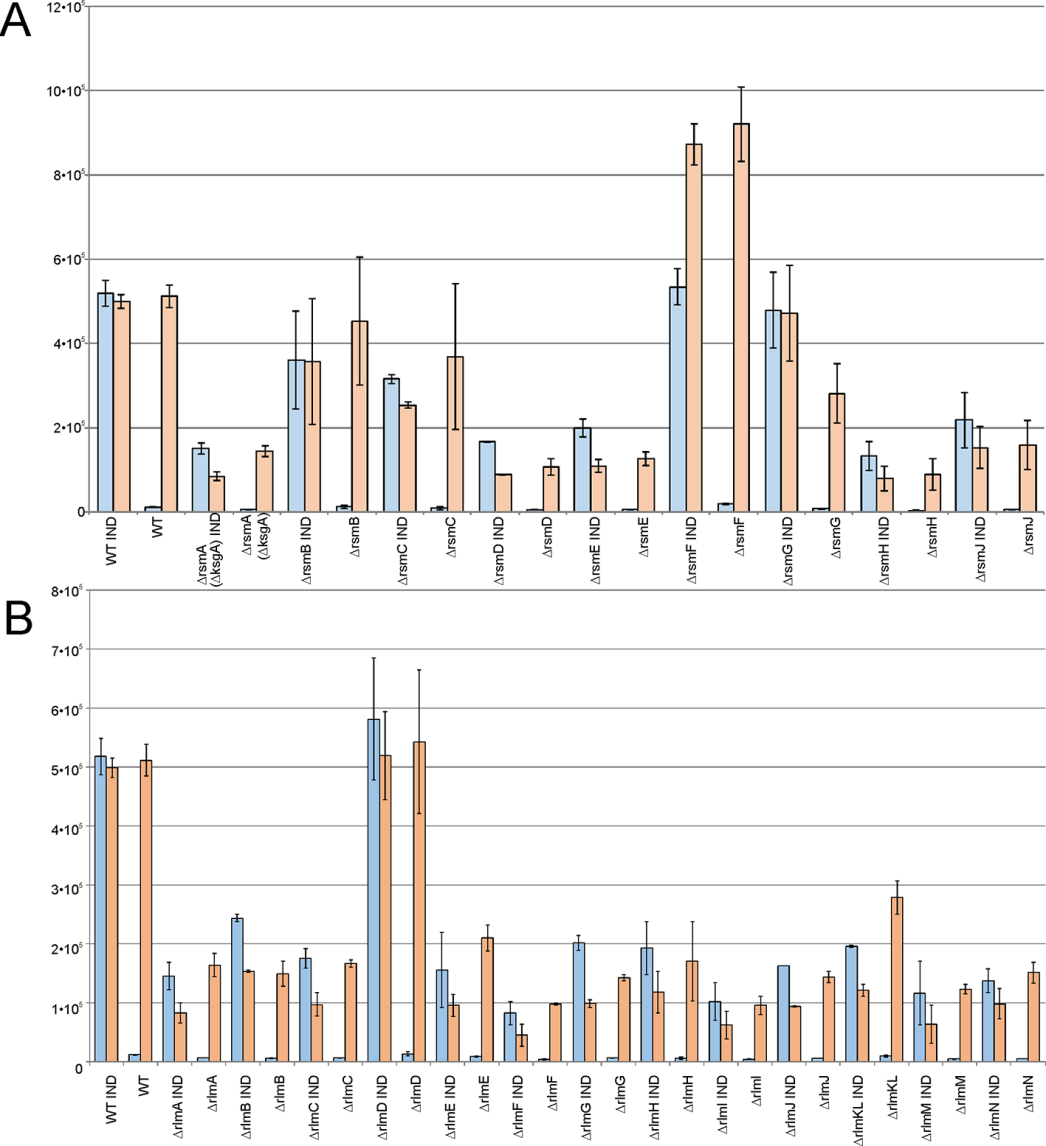
Influence of rRNA methyltransferase gene inactivation on the expression of exogenous reporter genes encoded on the plasmid carrying red fluorescent protein (RFP) gene under the control of a constitutive T5 promoter and cerulean fluorescent protein (CER) gene under a control of an induced Tet promoter. Shown are the intensities of the CER (blue) and RFP (orange) fluorescence in the overnight cultures of the strains devoid of the 16S rRNA **(A)** and 23S rRNA **(B)** methyltransferase genes as labeled below the graphs. WT corresponds to the parental isogeneic strain carrying all set of rRNA methyltransferase genes. IND marks the graphs corresponding to the induction of CER gene expression by anhydrotetracycline.

In a living cell, different mRNA species compete for the protein biosynthesis machinery. If ribosomes and other components of the translation apparatus are present in excess over the total mRNA, then the transcriptional control of gene expression might function optimally and an increase in certain mRNA synthesis would not limit the expression of other genes. However, if the number of active ribosomes is low or they are functioning inefficiently, then excessive transcription of a gene would have a negative influence on other genes’ expression due to the competition. To evaluate the influence of rRNA methylation on the availability of protein biosynthesis machinery for exogenous protein synthesis, we used a set of rRNA methyltransferase knockout strains transformed with a plasmid encoding for RFP under the constitutive T5 phage promoter and the *cer* gene under the inducible Tet promoter. The expression level of both genes could be monitored simultaneously in the rRNA MT knockout cells and compared to that of the wild-type cells. Thus, the overall ability of cells to support excessive protein synthesis might be deduced from this experiment as well as the influence of induced gene expression on the expression level of other genes, exemplified by a constitutively expressed *rfp* gene (**Figure 5**). The wild-type cells efficiently expressed both fluorescent proteins, and no reduction in constitutively transcribed *rfp* gene expression was observed upon additional expression of the *cer* gene. The yield of exogenous reporter protein synthesis was reduced for rRNA knockout strains. Only for the Δ*rsmB*, Δ*rsmC*, Δ*rsmG*, and Δ*rlmD* strains this reduction was mild, and not exceeding twofold. More significant, up to a 10-fold reduction of RFP expression was attributed to the lack of the remaining rRNA methyltransferases (**Figure 5**). Of note, a reduction in constitutively transcribed *rfp* gene expression was observed upon induction of *cer* gene transcription in the strains deficient in *rsmA*(*ksgA*)*, rlmA, rlmC*, *rlmE*, *rlmF, rlmG*, *rlmJ*, *rlmKL*, *rlmM*, and *rlmN*. The only exception in this rule is the Δ*rsmF* strain supporting twofold higher expression of the RFP gene, relative to that of the wild-type strain, as well as mildly increased expression of the CER gene, relative to that in the wild-type strain at the uninduced state, and upon CER gene induction. To be sure that this observation was well reproduced, we repeated this experiment in 24 independent cell culture replicates (**Figure S1**). While the absolute level of CER and RFP fluorescence varied from culture to culture, the tendency always remained the same. In the strain devoid of RsmF, both CER and RFP levels were higher when those in the wild-type strain. To check whether this increase in expression is due to transcription or translation enhancement, we compared the amounts of *cer* and *rfp* mRNAs (**Figure S2**). An increase in the yield of both *cer* and *rfp* gene transcription in the Δ*rsmF* strain shows evidence that the overall upregulation of CER and RFP synthesis in this strain could be explained, at least partially, by the upregulation of transcription.

Our findings support a hypothesis that in the majority of strains lacking particular types of rRNA methylation, ribosome availability became a limiting factor for gene expression, where the strain devoid of RsmF methyltransferase appeared to be an exception.

### Single Cell Analysis of the Efficiency of Protein Synthesis Upon the Inactivation of rRNA Methyltransferase Genes

**Figure 6:**
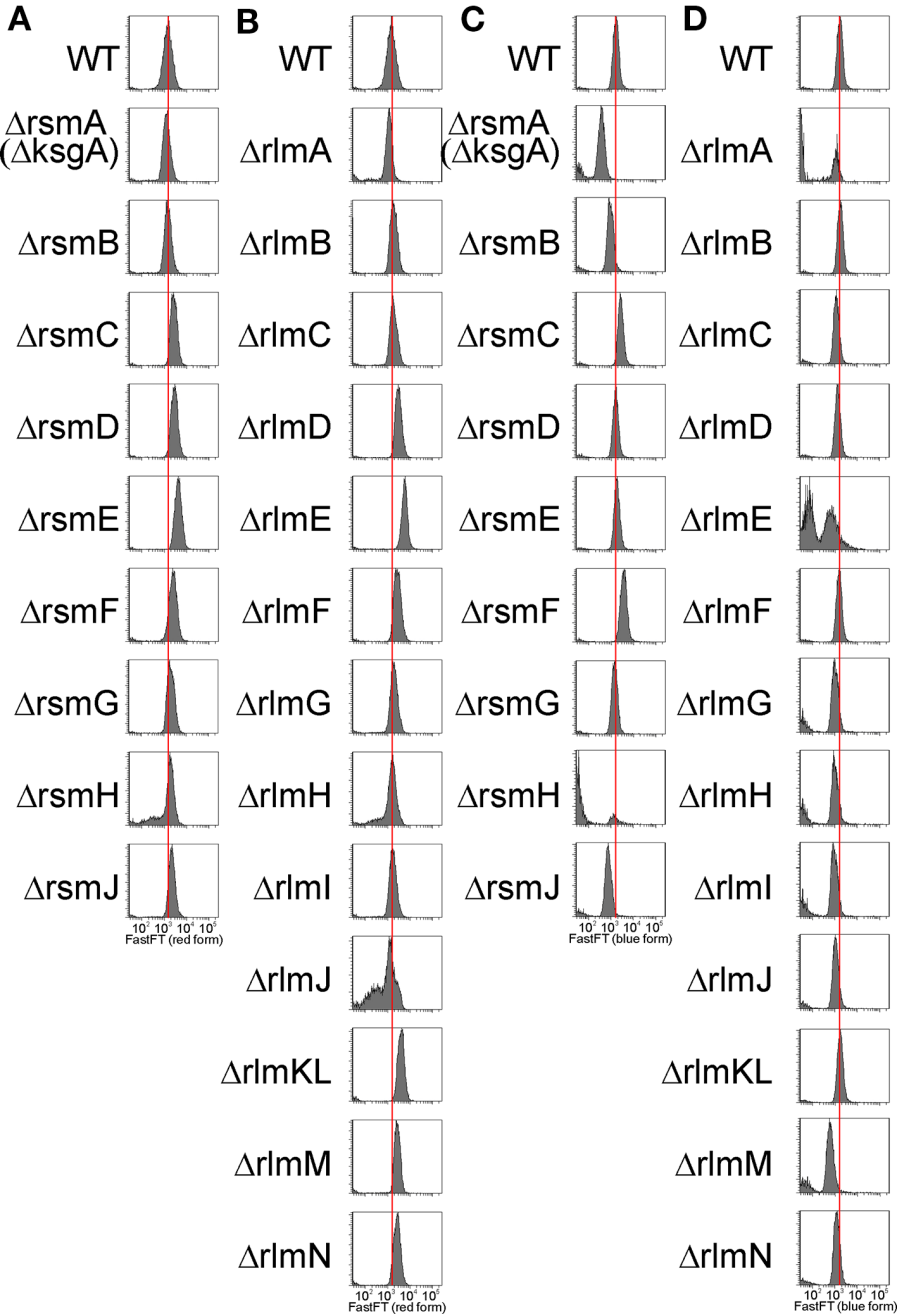
Influence of rRNA methyltransferase gene inactivation on the expression of exogenous reporter gene FastFT. Shown is a distribution of cells (arbitrary units) by the red fluorescence intensity corresponding to the completely mature FastFT form in overnight cultures of the strains devoid of the 16S rRNA **(A)** and 23S rRNA **(B)** methyltransferase genes as labeled on the left side of the graphs. Distribution of cells (arbitrary units) by the blue fluorescence intensity corresponding to the newly synthesized FastFT form in the cultures at the exponential growth (5 h post dilution) of the strains devoid of the 16S rRNA **(C)** and 23S rRNA **(D)** methyltransferase genes as labeled on the left side of the graphs.

To evaluate the capacity for exogenous protein synthesis in the individual cells of rRNA MT knockout strains, we applied a reporter plasmid encoding a FastFT fluorescent timer protein (Subach et al., 2009) and detected the intensity of fluorescence by the cell sorter. The FastFT protein undergoes a two-step maturation process in a way that a rapid formation of a blue fluorophore is followed by a slow conversion to a red fluorescent form with a half conversion rate of 7 h. With this rate, stationary phase cells, after 24 h of growth, contain mainly the fully converted red fluorescent form of FastFT, while rapidly growing cells contain the blue fluorescent form of the protein. We transformed rRNA MT knockout strains systematically with the plasmid coding for the FastFT protein under a control of an arabinose-inducible promoter and monitored the fluorescence at 405/460 nm for a newly synthesized blue fluorescent form and at 555/610 nm for a fully converted red fluorescent form (**Figure 6**). Monitoring of the FastFT protein level and synthesis was done throughout the growth curve of the bacterial culture.

At the stationary phase of the bacterial culture, the level of the predominant red form of the FastFT protein for the majority of the rRNA MT knockout strains exceeded that of the wild type (**Figures 6A, B**). We hypothesize that this might be due to the increase of the average cell size, rather than a change in FastFT protein biosynthesis. An exceptional presence of the blue FastFT form in the stationary phase cells was documented for the strain lacking the *rsmF* gene (**Figure S3**), which corresponds to a moderate increase in both CER and RFP reporter protein levels in this strain, relative to the wild-type strain described in the preceding section.

In the exponential growth phase (**Figures 6C, D**), the level of the predominant blue form of the FastFT protein was generally lower for the rRNA MT knockout strains, while the cell size was approximately the same. This is indicative of the decrease in exogenous protein synthesis efficiency upon rRNA MT gene inactivation, which is especially prominent in *rsmA*(*ksgA*), *rsmJ*, *rlmE*, and *rlmM* knockout cells. These findings correspond well with our data on the synthesis of CER and RFP reporter proteins discussed in the preceding section. Furthermore, a strain lacking the *rsmF* gene, and additionally the Δ*rsmC* strain, demonstrated a higher amount of the freshly synthesized blue FastFT protein form in the exponentially growing cells, in line with an increased expression of other exogenous fluorescent protein genes.

### Influence of rRNA Methyltransferase Gene Inactivation on the Composition of the Total Proteome

Lack of m^2^G966/m^5^C967 16S rRNA nucleotide modification perturbed the proteome of the cell and resulted in the misregulation of gene expression control by transcription attenuation (Prokhorova et al., 2013). Next, we sought to compare the proteomes of the strains lacking rRNA MT encoding genes. Proteins, whose abundance was changed in comparison with the parental strain, were detected by 2D protein gel electrophoresis (**Figure S4**). Surprisingly, none of the single rRNA methyltransferase gene knockouts led to a significant and reproducible perturbation of the proteome as revealed by 2D protein gel electrophoresis.

Three strains were selected for more detailed proteome analysis using a label-free shotgun proteome technique. The Δ*rlmE* strain demonstrated the most severe growth retardation, accumulation of ribosome assembly intermediates, and a significant reduction in the efficiency of reporter protein synthesis. The Δ*rsmF* strain was unique in its ability to support higher expression of the exogenous protein than the parental wild-type strain. The strain lacking the *rlmC* gene had a mild phenotypic abnormality and an increased level of the 17S rRNA precursor at low temperature. Very few proteins changed their abundance in the Δ*rlmC* strain (**Table 2**, **Table S1**). An analysis of transcription factors that might co-regulate genes whose expression depended on the RlmC revealed σS and ppGpp that regulate *sra* and *gadB* expression, although the statistical support is insufficient (Keseler et al., 2013). A more profound change was observed in the *rlmE* gene knockout. Visual inspection of the results suggests that σS, ppGpp, AppY, ArcA, and FNR activated genes are downregulated in the Δ*rlmE* strain, while genes repressed by PepA, ArcA, FNR, arginine, methionine, and pyrimidine nucleotides are upregulated. This statement, however, could get sufficient statistical support only for the transcription factor PepA (pvalue = 0.02, Bonferroni correction) (Keseler et al., 2013). Visual analysis of the genes whose expression was changed by inactivation of the *rsmF* gene revealed the possible involvement of Crp and σS, although the statistical support is insufficient (Keseler et al., 2013), and further partial verification with the help of RT qPCR (**Figure S5**) could not support the possible involvement of Crp in the response to the *rsmF* gene knockout. Notably, σS is among the proteins whose amount was decreased more than twofold upon RsmF inactivation. This may suggest that increased transcription of σ70-dependent reporter genes in this knockout strain might to some extent be explained by reduced competition over the RNA polymerase core enzyme. GO term analysis of the proteins whose abundance decreased upon RsmF rRNA methyltransferase inactivation revealed a TCA cycle (pvalue = 0.001). We found a certain degree of similarity between proteomic responses to *rlmC*, *rlmE*, and *rsmF* gene inactivation (**Table 2**). Interestingly, all three strains have a reduced concentration of Sra and GadB proteins.

**Table 2:** Significant differences in the proteome of *E. coli* caused by inactivation of *rlmC, rlmE*, and *rsmF* genes as revealed by panoramic proteome analysis.

Δ*rlmC*		Δ*rlmE*		Δ*rsmF*	
sra	0,28	gadB	0,21	astC	0,07
gadB	0,39	sra	0,23	acs	0,10
can	0,44	hyaB	0,30	modA	0,22
ytfE	2,05	cydA	0,31	pspE	0,22
		ybgS	0,40	ugpB	0,23
		appA	0,42	argT	0,25
		narG	0,44	sra	0,25
		mdtE	0,45	grcA	0,26
		glpQ	0,47	sdhA	0,27
		rpsU	0,48	msrB	0,28
		psiF	0,49	sdhB	0,30
		rpsL	0,50	aldA	0,31
		can	0,50	fdoH	0,32
		ansB	0,50	flu	0,33
		fadI	2,07	sthA	0,33
		nlpA	2,07	gadB	0,34
		fumA	2,14	gadC	0,34
		yfeX	2,14	sucB	0,35
		mdaB	2,21	yciF	0,36
		carA	2,21	psiF	0,36
		pck	2,26	putA	0,37
		carB	2,58	dadX	0,37
		metK	2,92	ynfK	0,37
		pyrI	3,22	osmY	0,39
		ydeN	3,32	ydfZ	0,39
		nanA	3,41	yhhA	0,41
		gdhA	3,52	yciE	0,41
		oppA	5,47	acnA	0,42
		ompF	6,86	rpoS	0,43
				yaiE	0,44
				frdB	0,44
				ybgS	0,44
				ynjE	0,45
				ompX	0,45
				tsx	0,46
				sucA	0,46
				hisJ	0,46
				iadA	0,47
				glcB	0,47
				mglB	0,48
				ftnA	0,48
				ybeL	0,49
				bfr	0,49
				sodC	0,49
				glnH	0,50
				nanA	2,03
				gatY	2,07
				miaB	2,09
				guaB	2,18
				iscS	2,19
				cysH	2,25
				metK	2,32
				cydA	2,35
				yidB	2,99
				ybeD	3,51
				mdaB	3,58
				oppA	3,61

Only proteins whose amount was changed more than twofold are listed. Complete analysis is available as **Table S1**.

### Phylogenetic Distribution of Orthologs of *E. coli* rRNA Methyltransferases

**Figure 7:**
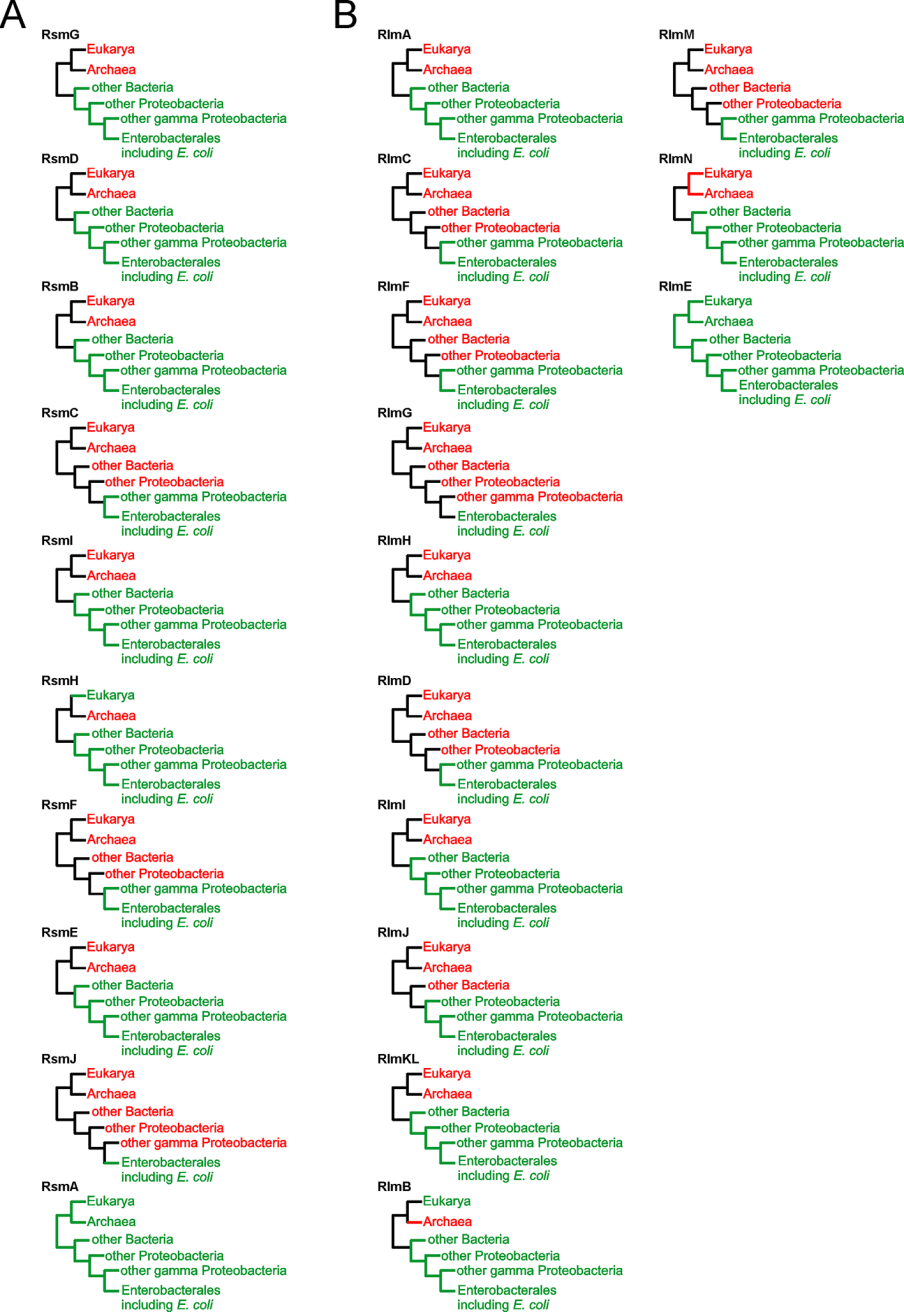
Phylogenetic distribution of the *E. coli* 16S rRNA **(A)** and 23S rRNA **(B)** methyltransferases orthologs. Shown are the simplified phylogenetic trees illustrating the occurrence of rRNA MT orthologs in the taxons beyond Enterobacteriales, gamma Proteobacteria, Proteobacteria, and Bacteria as well as in Eukarya and Archaea. Schematic trees are labelled by protein designations. Branches colored green correspond to those that contain orthologs of the methyltransferase, while those colored red do not.

Conservation of a protein may be a proxy of its functional importance. To compare the phylogenetic distribution of the complete set of *E. coli* rRNA methyltransferases, we performed blast searching (Altschul et al., 1990) for their orthologs. We aimed to determine a minimal phylogenetic group still containing orthologs of a particular *E. coli* rRNA methyltransferase. To this end, we verified whether any orthologs of a given *E. coli* rRNA methyltransferase might be identified outside Enterobacteriales, gamma Proteobacteria, Proteobacteria, and Bacteria, and whether any orthologs could be found in Eukarya and Archaea (**Figure 7**). We intentionally decided not to determine the entire phylogenetic trees of all rRNA methyltransferase families as this might be the matter of a separate, more focused, study.

The range of phylogenetic distributions in our set varied from universality for RsmA(KsgA) and RlmE, to extreme specificity to Enterobacteria for RsmJ and RlmG. Most frequently rRNA methyltransferase orthologs were found to be conserved among bacteria. For several *E. coli* rRNA methyltransferases, close paralogs were identified, such as RlmCD methyltransferases of gram positive bacteria for RlmC and RlmD (Desmolaize et al., 2011) or orthologs of RsmB in a BLAST search for RsmF homologues.

## Discussion

Several roles have been suggested for methylated rRNA nucleotides (Sergiev et al., 2011; Sergiev et al., 2018), among which ribosomal subunit assembly control appears amongst the most obvious ones. RsmA(KsgA) methyltransferase was shown to fulfill quality control over the last stages of small subunit assembly (Connolly et al., 2008), in line with a similar role of its eukaryotic homologue, Dim1, in ribosome biogenesis (Lafontaine et al., 1995). The mechanism of RsmA(KsgA)’s action suggested by Connolly et al. (2008) involved binding of the small subunit assembly intermediates by RsmA(KsgA), while its methyltransferase activity is delayed until the late stages of assembly. This type of activity might require more RsmA(KsgA) molecules relative to other rRNA MTs, which might cycle faster. Accordingly, we see the highest level of RsmA(KsgA) mRNA relative to mRNA coding for other rRNA MTs. We did not observe significant accumulation of assembly intermediates in the Δ*rsmA*(Δ*ksgA*) strain by sucrose gradient centrifugation, in line with earlier studies (Connolly et al., 2008). However, in consort with that work, we found an increase in the amount of the 17S rRNA precursor upon growth of the Δ*rsmA*(Δ*ksgA*) strain at a low temperature (**Figure 3**). Ubiquitous phylogenetic distribution of KsgA orthologs (**Figure 7A**) is explained by their involvement in the assembly of bacterial (Connolly et al., 2008) and eukaryal cytoplasmic (Lafontaine et al., 1995), as well as mitochondrial (Metodiev et al., 2009), small ribosomal subunits.

In addition, a significant amount of the 17S rRNA precursor accumulates in the Δ*rsmB* strain (**Figure 3**). RsmB is known to act on the early assembly intermediate of the 30S ribosome subunit, prior to the incorporation of the S19 protein (Weitzmann et al., 1991). It might be suggested that RsmB accelerates the assembly or prevents misassembly. Orthologs of RsmB are distributed among bacteria (**Figure 7A**), while in archaea and eukarya the same small subunit rRNA loop is modified to form acp^3^U (Kowalak et al., 2000) or m^1^acp^3^ψ (Wurm et al., 2010; Meyer et al., 2016) respectively. Enzymes Nep1 (Wurm et al., 2010) and Tsr3 (Meyer et al., 2016), responsible for the formation of the m^1^acp^3^ψ nucleotide, are documented to serve a role in ribosome assembly and might be hypothesized to have a function reminiscent to that of RsmB.

Unexpectedly, inactivation of the 23S rRNA methyltransferase RlmC, responsible for the formation of m^5^U747, also resulted in the accumulation of the 17S rRNA precursor (**Figure 3**). This phenomenon is not unique. Inactivation of the large subunit pseudouridine synthase RluD also resulted in the accumulation of the 17S rRNA precursor (Gutgsell et al., 2005), although later this phenotype was found to be dependent on the combination of the *rluD* knockout and *E. coli* K12-specific RF2 allele carrying threonine at the position 246 (O’Connor and Gregory, 2011). It seems likely that an influence of 23S rRNA methylation by RlmC on the processing of the small subunit rRNA is indirect, similar to that of RluD.

A severe assembly defect was known to be associated with inactivation of the RlmE methyltransferase (Bugl et al., 2000; Caldas et al., 2000; Hager et al., 2002; Arai et al., 2015). Here we corroborated and extended previous studies (**Figure 2**), demonstrating that while *rlmE* inactivation leads to a severe manifestation of ribosome misassembly, other rRNA MT knockouts have a marginal, if any, effect on the accumulation of ribosome assembly intermediates which might be distinguished by sucrose density centrifugation. In line with this, *rlmE* inactivation leads to the most significant growth retardation among the other rRNA MT gene knockouts. The severe phenotype of *rlmE* gene inactivation goes in parallel with the universal phylogenetic distribution of its orthologs (**Figure 7B**). A number of genes whose expression is dependent on σ^S^ and ppGpp, as well as ArcA and FNR, were found downregulated in the Δ*rlmE* strain (**Table 2**). This result may indicate lagging of the growth phase of the Δ*rlmE* strain relative to that of the wild type, which may also result in higher oxygenation of the bacterial culture sensed by ArcA and FNR. RlmC inactivation, which moderately affected the growth rate of bacteria, resulted in decreased expression of *sra* and *gadB* genes. Both these genes were similarly downregulated in the strains deficient in the *rlmE* and *rsmF* genes. This phenomenon might also reflect a lag in the growth phase of rRNA MT knockout cells, since both *sra* and *gadB* genes are normally expressed in the stationary phase (Castanie-Cornet and Foster, 2001; Izutsu et al., 2001).

Another common assumption is that rRNA modification might be functional in a particular set of conditions. In such a case, it might be anticipated that the expression of the corresponding rRNA MT genes might be regulated in a condition-dependent manner. Analysis of rRNA MT gene expression throughout the bacterial growth cycle revealed that the predominant majority of rRNA MT genes is significantly upregulated in the exponential growth phase (**Figure 1**), along with the expression of rRNA operons. These data generally support a previous work (Sergiev et al., 2012) performed on the basis of GEO database analysis. Among the rRNA MT genes, *rlmE* and *rsmB* seem to be induced at the later growth phase, demonstrating a distinct way of transcriptional control. *RlmE* is known to be under heat shock control (Bugl et al., 2000), while the mechanism of *rsmB* gene regulation remains unknown. Expression of the predominant majority of rRNA MT in the exponential growth phase does not support a function of methylated rRNA nucleotides within specific conditions.

Protein biosynthesis is one of the major resource consuming processes. Suboptimal protein biosynthesis efficiency caused by a lack of rRNA methylation may become a significant problem if resources are additionally spent on the synthesis of a protein coded in an artificially expressed gene. In line with this assumption, the growth rates of the majority of rRNA MT knockout strains are almost indistinguishable from that of the wild-type strain, except for the *rlmE* knockout, leading to the accumulation of ribosome assembly intermediates. However, induction of an exogenous gene, exemplified in this study by *rfp* and *cer*, decreases the growth rates of the rRNA MT knockout strains more significantly than that of the parental strain containing a complete inventory of rRNA MT coding genes. Almost all rRNA MT knockout strains were able to support only ½ to ¼ of the wild-type expression level of the constitutively expressed *rfp* gene. Additionally, induction of *cer* gene expression in the majority of the rRNA MT knockout strains not only failed to reach the wild-type expression levels, but also led to a more significant decrease in the expression of the *rfp* gene. This tendency is well reproduced by the evaluation of protein biosynthesis efficiency at a single cell level by flow cytometry. The likely interpretation of this fact is a mildly reduced protein synthesis capacity of the strains devoid of rRNA MT genes, which becomes a significant problem if a cell is under the burden of the synthesis of an exogenous protein. Among the rRNA MT deletion strains, the most compromised in the ability to synthesize exogenous proteins are the Δ*rlmA*, Δ*rlmG*, Δ*rlmH*, Δ*rlmI*, Δ*rlmJ*, Δ*rlmM*, Δ*rlmN*, Δ*rlmE,* Δ*rsmA*(Δ*ksgA*), Δ*rsmH,* and Δ*rsmJ* strains.

Apart from the general tendency, *rsmF* gene knockout resulted in a moderate increase of exogenous gene expression compared to the wild type. RsmF methyltransferase is responsible for the modification of m^5^C1407 of the 16S rRNA (Andersen and Douthwaite, 2006), which increases the aminoglycoside sensitivity of *E. coli* (Gutierrez et al., 2012). The molecular mechanism of a small increase in exogenous gene expression in the Δ*rsmF* strain is unknown; a likely explanation is the suboptimal functioning of some regulatory mechanism. The transcriptional upregulation of reporter gene expression might be involved, at least partially. According to the proteome analysis, *rsmF* knockout leads to an alteration in the abundance of a large number of proteins, including decreased amounts of σ^S^ and downregulation of a number of genes transcribed with the help of this sigma factor. Perhaps, the increased propensity of the *rsmF* knockout strain to synthesize an exogenous protein might reflect a secondary effect.

This work might be of particular interest, as it is the first comprehensive study of all rRNA methyltransferase knockouts. Although only a few rRNA MT knockouts were found to have an influence on bacterial growth, ribosome assembly, or proteome, the majority of the knockout strains demonstrated a suboptimal capability to synthesize exogenous proteins. This may indicate a necessity of rRNA methylation at increased loads on the protein biosynthesis apparatus. More methods are needed to assess the subtle advantages of rRNA methylation in bacteria.

## Materials and Methods

Homology search was done with BLAST (Altschul et al., 1990), using the model organisms database. The resulting phylogenetic distribution of the BLAST hits was used to deduce whether any orthologous proteins could be found beyond Enterobacteriales, gamma Proteobacteria, Proteobacteria, and Bacteria, and whether any orthologs could be found in Eukarya and Archaea. Visual inspection of the search results was used to filter out paralogous, rather than orthologous proteins. In doubtful cases, a reciprocal BLAST search, with a putative orthologous protein identified in the original search, was performed to check whether the protein used as bait for the initial search would be found as its closest homologue among the proteins of *E. coli*.

In all experiments, the strains *ΔrsmG (JW3718), ΔrsmD (JW3430), ΔrsmB (JW3250), ΔrsmC (JW4333), ΔrsmH (JW0080), ΔrsmF (JW5301), ΔrsmE (JW2913), ΔrsmJ (JW5672), ΔrsmA(ksgA) (JW0050), ΔrlmA (JW1811), ΔrlmC (JW2756), ΔrlmF (JW5107), ΔrlmG (JW5513), ΔrlmH (JW0631), ΔrlmD (JW0843), ΔrlmI (JW5898), ΔrlmJ (JW3466), ΔrlmK/L (JW0931), ΔrlmB (JW4138), ΔrlmM (JW2777), ΔrlmN (JW2501), and ΔrlmE (JW3146)* from the Keio collection (Baba et al., 2006) were used and compared with the parental wild-type strain BW25113 (Datsenko and Wanner, 2000).

For the rRNA MT expression analysis, an overnight culture of wild-type E. coli was diluted in triplicate in fresh LB media to A260 0.01 and grown at 37°C in a shaker. Aliquots of cells were removed at 1, 2, 3, 4, 5, 6, 7, and 56 h and used for total RNA purification with Trizol reagent (Invitrogen), followed by cDNA synthesis with either a Maxima First Strand cDNA Synthesis Kit for RT-qPCR (Thermo) with a random hexamer primer or a Superscript reverse transcriptase (Invitrogen). Quantitative PCR was performed by a Maxima Hot Start DNA polymerase (Thermo) in the presence of SYBR green. The following primers were used for amplification of indicated mRNAs: rsmA(ksgA) (CCCTTTTGCGGGTTAATGGC and ACGCTTCGGGCAAAACTTTC), rsmB (CTATGCCACCTGTTCGGTGT and CTGTTTCGCAAAGTTCGGCA), rsmC (GCGCATAATCTGCCAGCATCand AAGAACAAACCGGAAGCCCA), *rsmD* (CGCAAAAAGGTACACCGCAT and CAGCCAGCCGTTATCTTCCA), *rsmE* (TGAGCAGTGTGGTCGTAACCand ACCGGTAACGGCAACGTATT), *rsmF*(CCGATTTTCTCGGTTGGGGA and ATACCACTCCTCCGCTTCCT), *rsmG* (GGACGCACGATAGAGAGTGGand TTCGGTCCGCGATCCTAATG), *rsmH* (CTCACGTCTGATCCTCTCGC and CAATAGTCTTCGCAACGGCG), *rsmJ* (AATTCCAGATGTTCCGGCGT and TGCCTTATCTGTTCTGGCGG), *rlmA* (AAATCAGCCCCTTCAGCTCC and CCGATACCAGTATGGACGCC), *rlmB* (GCCAGGACGTCAGTATCAGG and AGGATCAGCAGGAACGGTTG), *rlmC* (GGGCTTTGGTTTACACTGCG and AAACTGAGTGGAGTCCAGCG), *rlmD* (TCGACAATGTCACTGGAGCC and GATGTTCCCTGGGGCTATCG), *rlmE* (AGGTCGACAACCGTCATTCC and AAAGGGGTTACGTTCCCGTG), *rlmF* (CATCACAGCCGCTACGATCTand AAGTCTACGCTTTGCTCCCC), *rlmG* (AATGCCAGTGTTTTCGGCAC and ACGGGATTGATGAGTCGAGC), *rlmH* (TGCTCACCCTCTTTGTCGAG and TTTACCGAGTACCTGCGTCG), *rlmI* (ATCGCGATAAGTACGCAGCA and GAAGCGCTGGATATTGCACG), *rlmJ* (CAGTTAGGCAGCGAACATGC and CTGACCGCTACGGTTGAAGT), *rlmKL* (TTTGAAACGTCTGCTGCGTG and CAGGCCGTCGAGATCCATAC), rlmM (CTTCAACACGCAGTTCACCG and CATTTGCCGCCAGAAGATCG), *rlmN* (ATGTCGATGGCTTCACCCTG and TATCGATGCTGCCTGTGGTC), *oppA* (AATCGTTCTTGAACGCAGC and GATCAACGTGAACTTCGTCC), *metK* (AGGCTGAAGTGCGTAAAAAC and GGGCAGAATTGGCTTGATG), *nanA* (GTGGTGTACAACATTCCAGC and CGAAGATTTCGTCGTAACCG), *gatY* (TTTGCCATCGCTTTGATGTC and TGGCATACATCCCATGAGC), *guaB* (AAGACTTCCAGAAAGCGGAA and TTCACGGATACGTTGCAGTA), *mdaB* (CAGCGACTACGATGTCAAAG and TTTTCGACGGATCTTTGCG), *ybeD* (CAGGCGTTACCTGAGCTG and TTTGCCCAGTTCTTCATACAGT), *astC* (ATTGACGACTCTACCTGTGC and TCACGCCGTAGTGCATATAG), *acs* (GCAGTATTCCGCTGAAGAAA and GATCTTCGGCGTTCATCTCT), *modA* (GCCTGCGGATCTGTTTATTT and TTCAGCAGTGAAGTCCAGTT), *psp* (ATCGATGTTCGTGTTCCAGA and CCCATCTCGCTAAGGATCTC), *ugpB* (GCTGGATCCAACTGGAAAAC and TTCATCCTTACGACCGACG), *argT* (TACCGATAAACGTCAGCAGG and CCTTTACTACGCCAGGTCTC), *sra* (AATCGAACCGTCAGGCAC and TTTTCAGCGGGGCGTTT), *cer* (TGAGCAAGGGCGAGGAGC and TGGTGCAGATGAACTTCAGG), *rfp* (GCTGATCAAGGAGAACATGC and AGGATGTCGAAGGCGAAGG). Quantification of expression was done by ΔΔCt method using 16S rRNA as a reference (gAgAATgTgCCTTCgggAAC and CCgCTggCAACAAAggATAA for MT gene expression analysis or CATTGACGTTACCCGCAGAAGAAG and CTACGAGACTCAAGCTTGCCAGTA for other gene expression analyses). To estimate the proportion of 17S rRNA processing, an intermediate RT qPCR approach was used. The following primer sequences were used for the 16S rRNA (GAAGAGTTTGATCATGGCTCAG and CCACTCGTCAGCAAAGAAG) and for the 17S rRNA processing intermediate (TCATTACGAAGTTTAATTCTTTGAGCG and GAAGAGTTTGATCATGGCTCAG). The proportion 17S/(16S + 17S) was calculated by normalization of the levels of the 5′-end-extended 17S transcript to the total amount of 16S and 17S transcripts.

To assess the accumulation of assembly intermediates, cells of rRNA MT knockout strains and a BW25113 strain (WT) and were grown in 500 ml of an LB medium at 37°C or 20°C to A_600_ 0.6, slowly cooled on ice, and harvested by centrifugation. Cells pellets were resuspended in a lysis buffer (20 mM HEPES-KOH pH 7.5, 4.5 mM Mg(OAc)_2_, 150 mM NH_4_Cl, 4 mM β-mercaptoethanol, 0.05 mM spermine, 2 mM spermidine buffer) and lysed by ultrasonication. After removal of cell debris, lysates containing approximately 1,200 pmol of ribosomes were applied to either a 10% to 30% sucrose gradient in a buffer 20 mM HEPES-KOH pH 7.5, 1 mM Mg(OAc)_2_, 200 mM NH_4_Cl, 4 mM β-mercaptoethanol, or a 10% to 40% sucrose gradient in a buffer 20 mM HEPES-KOH pH 7.5, 10 mM Mg(OAc)_2_, 200 mM NH_4_Cl, 4 mM β-mercaptoethanol. Ultracentrifugation was performed by an SW41Ti rotor at 19,000 rpm for 19 h followed by optical density monitoring at 260 nm.

To create a pRFPCERtet construct, a pRFPCER plasmid (Osterman et al., 2013) was digested with HindIII and SacII and ligated with pair of pre-annealed complementary oligonucleotides (TetR F 5′ AGCTTGGGAAATCATAAAAAATTATTTGCTTACTCTATCATTGATAGAGTTATAATAGCCGC-3′ and TetR R 5′-GGCTATTATAACTCTATCAATGATAGAGTAAGCAAATAATTTTTTATGATTTCCCA-3′), containing a T5 promoter with the TetR binding site. The obtained plasmid was digested with SacII and NdeI restriction enzymes and ligated with pair of pre-annealed complementary oligonucleotides (5′-CACACAACAAAGGAGGTAC and 5′-TAGTACCTCCTTTGTTGTGTGGC), containing a highly efficient ribosomal binding site. The resulted plasmid was used for further study as pRFPCERtet.

To monitor growth rates upon exogenous gene overexpression, and to evaluate protein synthesis efficiency, cells of rRNA MT knockout strains and the BW25113 strain (WT) were transformed with the plasmid pRFPCERtet. Overnight cultures of the transformants, in triplicate for each strain, were diluted by LB with or without anhydrotetracycline 0.2 ug/ml to A_600_ 0.01 in a 96 well plate. Cells were cultivated with continuous shaking at 37°C with automatic A_600_ monitoring every 30 min by a Janus workstation (Perkin Elmer). Growth rates of rRNA MT knockout strains, and the wild-type strain not transformed by any plasmid, were measured likewise.

For evaluation of CER and RFP protein synthesis efficiency, cells transformed by the plasmid pRFPCERtet were grown in triplicates for 18 h in 200 ul LB media at 37°C with or without anhydrotetracycline 0.2 ug/ml in a 96 deep well plate with continuous shaking. After incubation, the cells were centrifuged in a 96 well plate and washed twice with 0.9% NaCl. The fluorescence of the cells was measured by a Victor X5 plate reader (Perkin Elmer) at 430/486 nm for CER and 531/595 nm for RFP.

To determine *in vivo* protein synthesis efficiency, the cells of the wild-type and rRNA MT knockout strains were transformed by a plasmid encoding the FastFT protein under a control of an araBAD promoter (Subach et al., 2009). Cells grown in LB media with 10 mM arabinose at 37°C after 48 h were diluted 1:100 by a fresh LB media with 10 mM arabinose. An aliquot was taken at various time points; cells were isolated by centrifugation, washed two times by sterile PBS, and analyzed by a fluorescently activated cell sorter BD FACSAria III at the wavelengths 405/460 nm and 555/610 nm.

Comparative proteome analysis using 2D PAGE was performed as described (Hoch et al., 2015). Not less than three independently grown cultures were used for each knockout strain.

Shotgun comparative proteome analysis was performed as described (Toprak et al., 2014; Osterman et al., 2015). Briefly, cells resuspended in 0,75% w/w RapiGest SF (Waters) were lysed by sonication. After debris removal, protein cysteine bonds were reduced with 10 mM dithiothreitol and alkylated with 30 mM iodoacetamide. Trypsin was added in a 1/50 w/w ratio trypsin/protein and incubated at 37°C overnight. To stop trypsinolysis, trifluoroacetic (TFA) acid was added to the final concentration of 0,5% v/v. Peptides were desalted and resuspended in 3% acetonitrile (ACN), 0.1% TFA, to a final concentration of 2 µg/µl. Mass spectrometry analysis was performed on a TripleTOF 5600+ mass-spectrometer with a NanoSpray III ion source (ABSciex, Canada) coupled to a NanoLC Ultra 2D+ nano-HPLC system (Eksigent). For protein identification,.wiff data files were analyzed with ProteinPilot 4.5 revision 1656 (ABSciex) using the Paragon 4.5.0.0 revision 1654 (ABSciex) search algorithm and a standard set of identification settings to search against SwissProt database, species *Escherichia coli*.

## Data Availability Statement

The proteomic data in this article is available at ProteomeXchange, accession: PXD017171.

## Author Contributions

SE contributed to the design of experiments, 2D protein gel electrophoresis, exogenous gene expression analysis with reporter construct, data analysis. VT, AP, and PP contributed to 2D protein gel electrophoresis and data analysis. PP, EG, AZ, MD, and AG contributed to sucrose gradient centrifugation, exogenous gene expression analysis with reporter construct and RT qPCR expression analysis. PP, EG, and AZ performed 17S precursor quantification. MR performed the flow cytometry experiments. IO contributed to exogenous gene expression analysis with reporter construct. MS, OP, and VG contributed to proteome analysis. AB, PS, and OD contributed to the design of the study. All authors contributed to the manuscript writing.

## Funding

This work was funded by the Russian Science Foundation grant 19-14-00043 in a part related to growth rate measurement, proteome analysis and RT qPCR, Russian Foundation for Basic Research grant 17-00-00366 in a part related to reporter constructs. Scholarships to the authors and equipment were provided in part by Lomonosov Moscow State University’s government funding and development program.

## Conflict of Interest

The authors declare that the research was conducted in the absence of any commercial or financial relationships that could be construed as a potential conflict of interest.
