# Supplementary material for: Comprehensive Functional Analysis of Escherichia coli Ribosomal RNA Methyltransferases
Source: Front Genet. 2020 Feb 27;11:97. doi: 10.3389/fgene.2020.00097 (PMC7056703; doi:10.3389/fgene.2020.00097)
Supplement: Supplementary file 1 [file DataSheet_1.docx]

Supplementary figures

Comprehensive functional analysis of *Escherichia coli* ribosomal RNA methyltransferases

Philipp Pletnev^1,2,3^†, Ekaterina Guseva^4^†, Anna Zanina^1^†, Sergey Evfratov^1^, Margarita Dzama^4^, Vsevolod Treshin^1^, Alexandra Pogorel’skaya^4^, Ilya Osterman^1,2^, Anna Golovina^5^, Maria Rubtsova^1,2^, Marina Serebryakova^2,5^, Olga V. Pobeguts^6^, Vadim M. Govorun^6^, Alexey A. Bogdanov^1,5^, Olga A. Dontsova^1,2,3,5^ and Petr V. Sergiev*^1,2,5,7^

^1^Department of Chemistry, Lomonosov Moscow State University, Moscow, Russia

^2^Center of Life Sciences, Skolkovo Institute of Science and Technology, Moscow, Russia

^3^Shemyakin-Ovchinnikov Institute of Bioorganic Chemistry, Moscow, Russia

^4^Faculty of Bioengineering and Bioinformatics, Lomonosov Moscow State University, Moscow, Russia

^5^Belozersky Institute of Physico-Chemical Biololgy, Lomonosov Moscow State University, Moscow, Russia

^6^Federal Research and Clinical Centre of Physical-Chemical Medicine, Moscow, Russia

^7^Institute of Functional Genomics, Lomonosov Moscow State University, Moscow, Russia

*Corresponding Author
[petya@genebee.msu.ru](mailto:petya@genebee.msu.ru)

†These authors have contributed equally to this work as Co-first authors

**Supplementary figures**

**
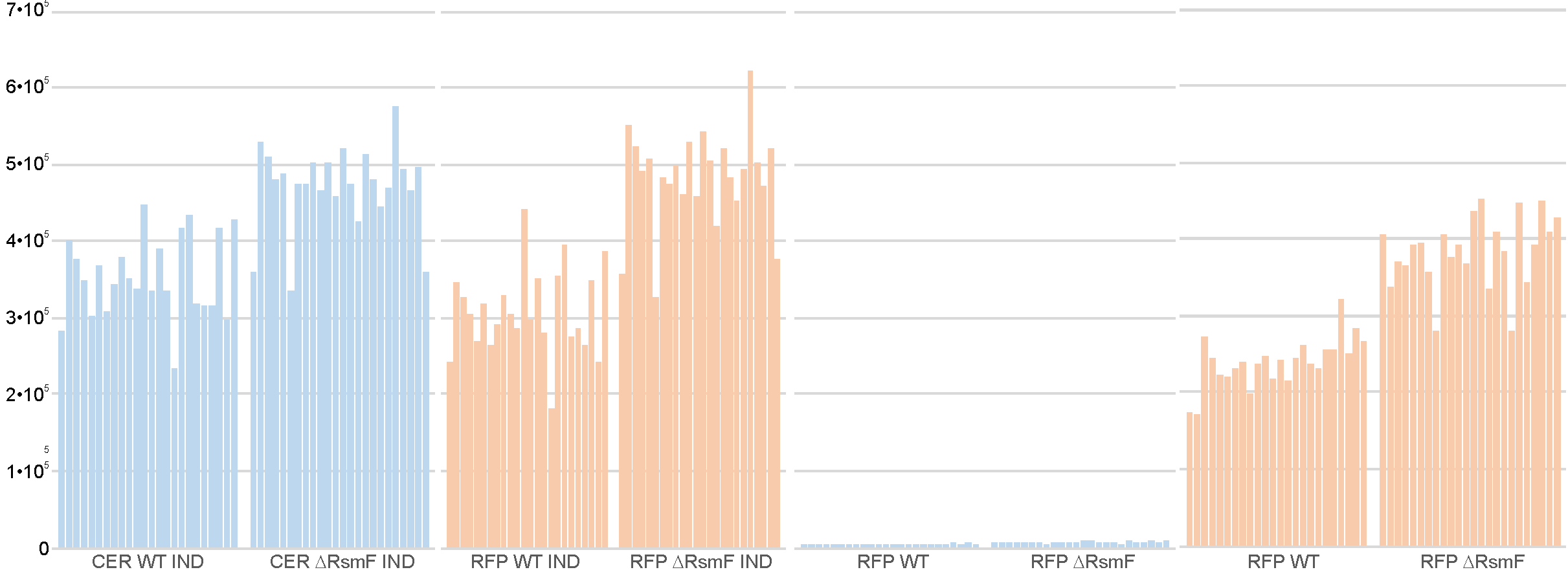
**

**Supplementary figure 1.** Influence of the rRNA methyltransferase *rsmF* gene inactivation on the expression of exogenous reporter genes encoded on the plasmid carrying RFP gene under the control of a constitutive T5 promoter and the CER gene under a control of an induced Tet promoter. Shown are intensities of the CER (blue) and RFP (orange) fluorescence in the 24 independent overnight cultures of the parental isogeneic strain (WT) and the strain devoid of *rsmF* (Δ*rsmF*). IND marks the graphs corresponding to the induction of CER gene expression by anhydrotetracycline.

**
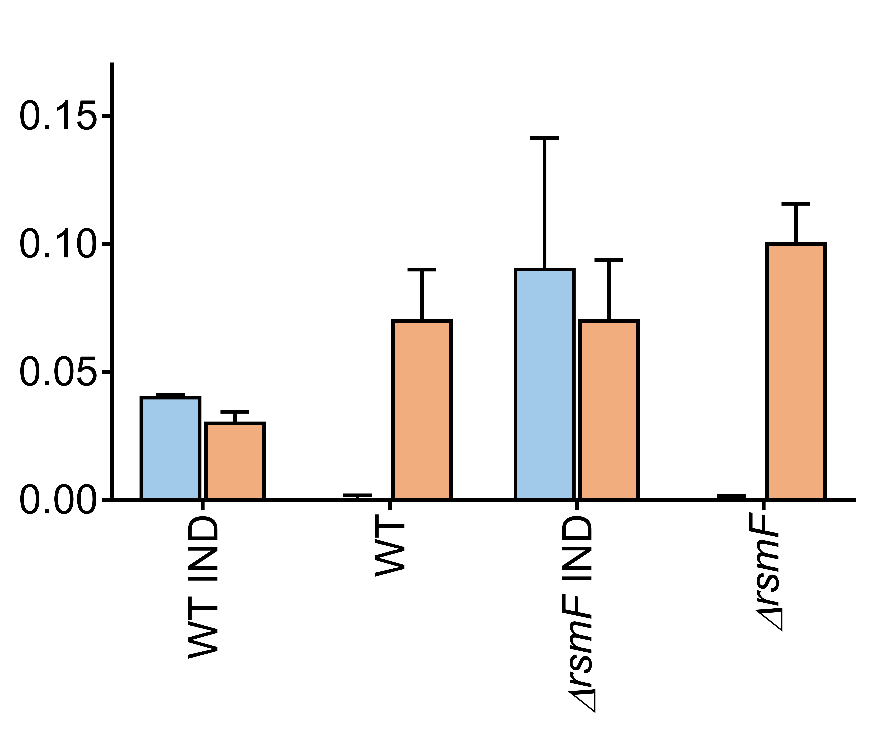
**

**Supplementary figure 2.** Influence of the rRNA methyltransferase *rsmF* gene inactivation on the level of mRNAs of exogenous reporter genes encoded on the plasmid carrying the RFP gene under the control of a constitutive T5 promoter and the CER gene under a control of an induced Tet promoter. Shown are relative amounts of the CER mRNA (blue) and RFP mRNA (orange) determined by RT qPCR and normalized to the level of the 16S rRNA in the exponential growth phase cultures of the parental isogeneic strain (WT) and the strain devoid of *rsmF* (Δ*rsmF*). IND marks the graphs corresponding to the induction of CER gene expression by anhydrotetracycline.

**A B**

**
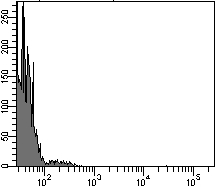

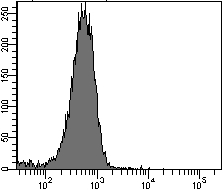
**

**Supplementary figure 3.** Influence of the rRNA methyltransferase *rsmF* gene inactivation on the expression of exogenous reporter gene FastFT. Shown is a distribution of cells (arbitrary units, ordinate) by the blue fluorescence intensity (log scale, abscissa) corresponding to the newly made FastFT form in the overnight cultures of the wild type (**A**) and Δ*rsmF* (**B**) strains.

**
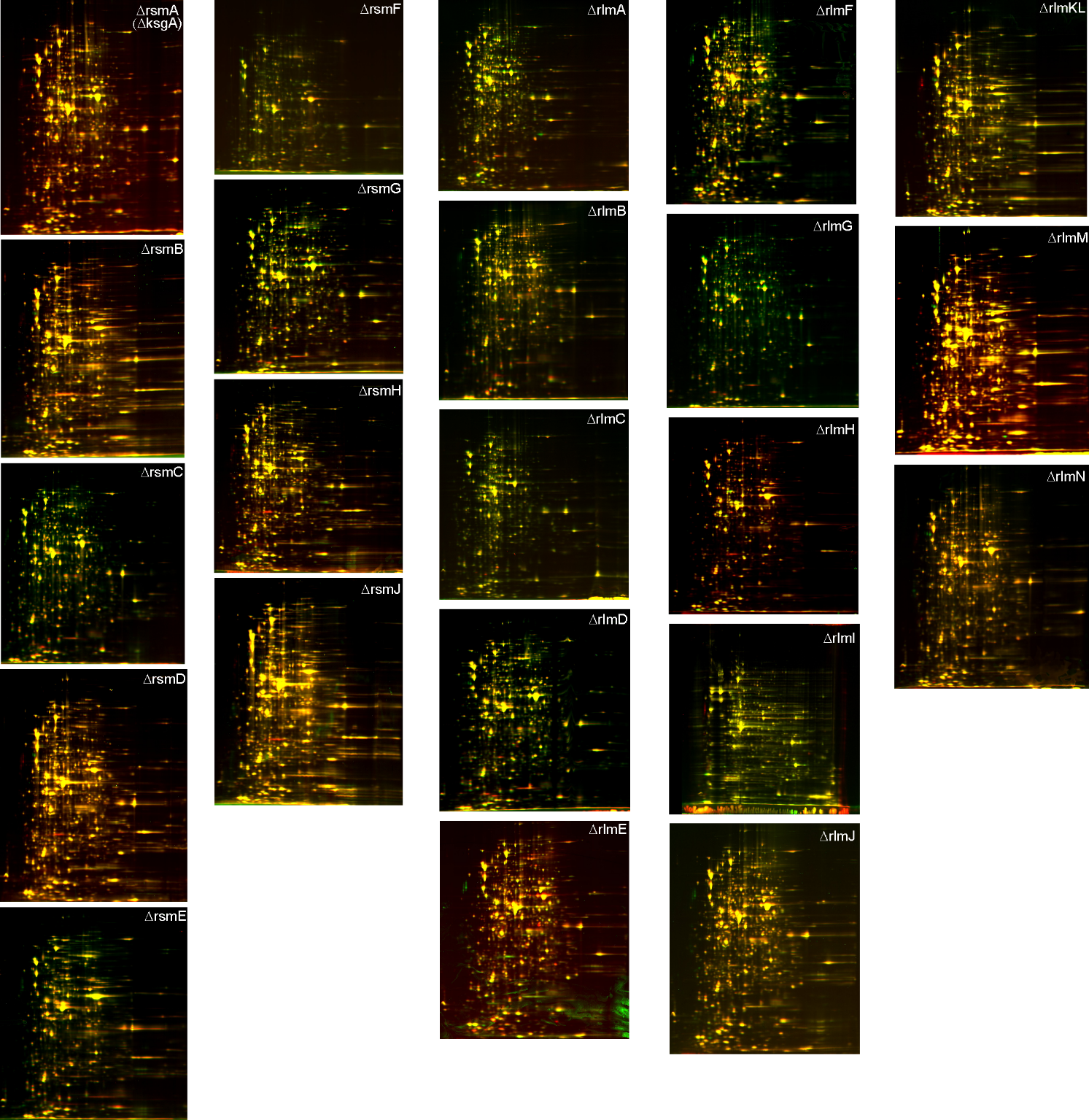
**

**Supplementary Figure 4.** Influence of rRNA methyltransferases genes inactivation on the *E. coli* cell proteome studied by differential 2D protein gel electrophoresis. The horizontal axis corresponds to the separation by isoelectric point, while the vertical axis corresponds to the separation by protein mass. Samples corresponding to the parental strain carrying all set of rRNA methyltransferase genes (wild type) are labelled by Cy3 dye visualized as green color, while samples corresponding to rRNA methyltransferase knockout strains are labelled by Cy5 dye visualized as red color. Yellow spots correspond to the proteins equally present in both types of samples. Knockout strains used are indicated in the top right corners of each panel.

| **Gene** | **RNA level Δ*rsmF*/WT** | **Standard deviation** | **protein level Δ*rsmF*/WT** 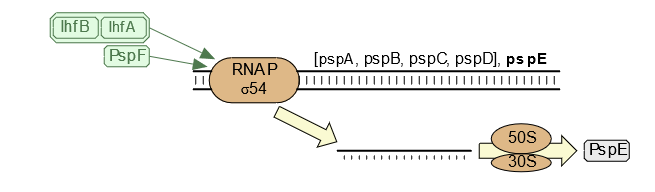 | **Regulation** |
| --- | --- | --- | --- | --- |
| **acs** | 0,3 | 0,4 | 0,1 | 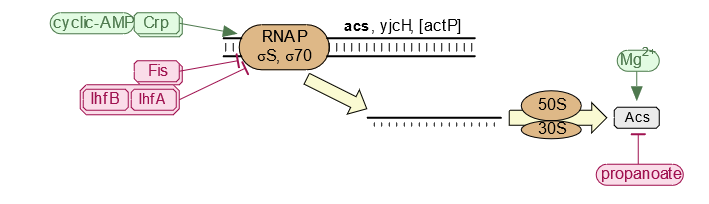 |
| **argT** | 0,7 | 0,5 | 0,25 | 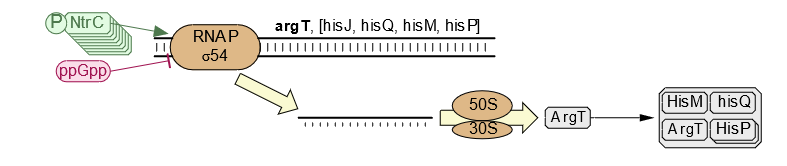 |
| **astC** | 0,3 | 0,3 | 0,07 | 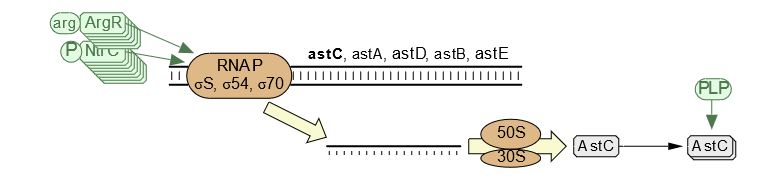 |
| **gatY** | 1,3 | 0,6 | 2 | 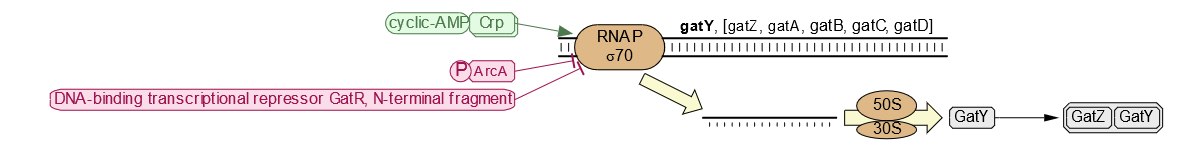 |
| **guaB** | 0,4 | 0,4 | 2,18 | 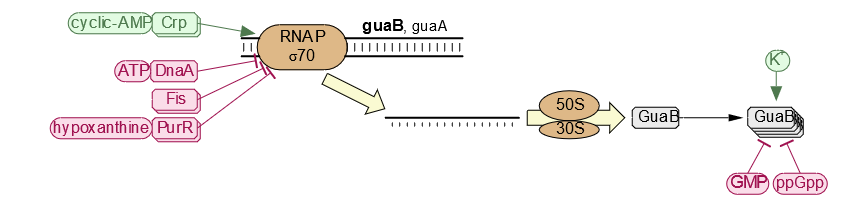 |
| **mdaB** | 1 | 0,6 | 3,6 | 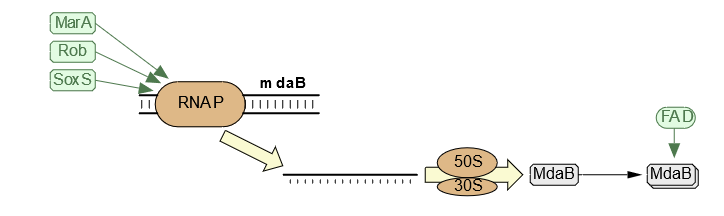 |
| **metK** | 0,6 | 0,5 | 2,32 | 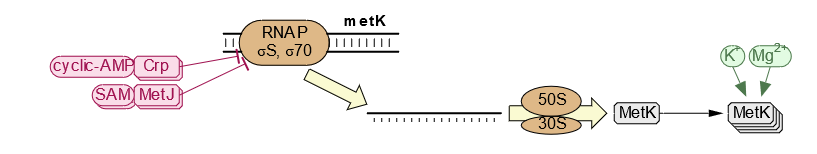 |
| **modA** | 1,5 | 0,9 | 0,22 | 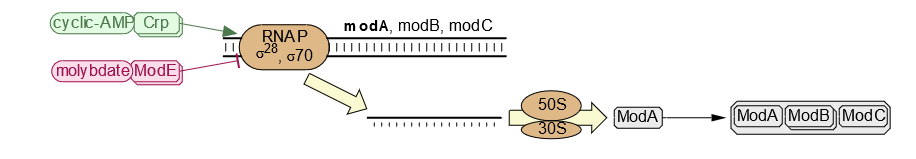 |
| **nanA** | 2,5 | 2,5 | 2,03 | 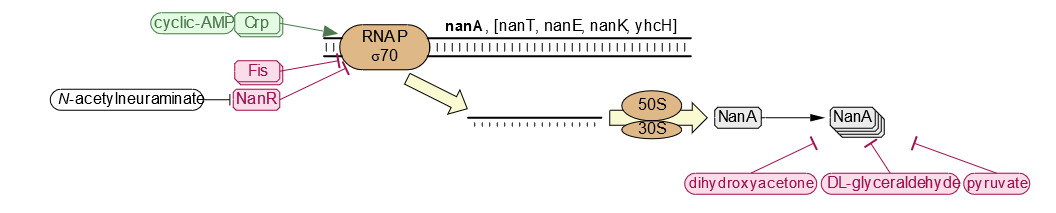 |
| **oppA** | 1 | 0,6 | 3,6 | 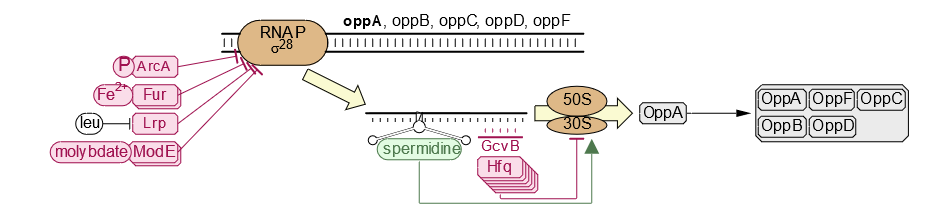 |
| **pspE** | 1,8 | 0,8 | 0,22 | 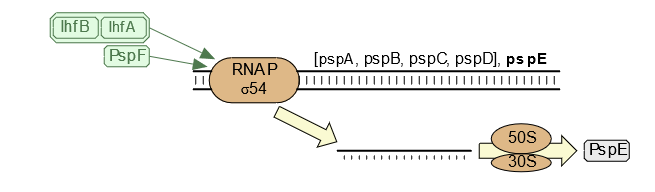 |
| **sra** | 1,3 | 1,1 | 0,25 | 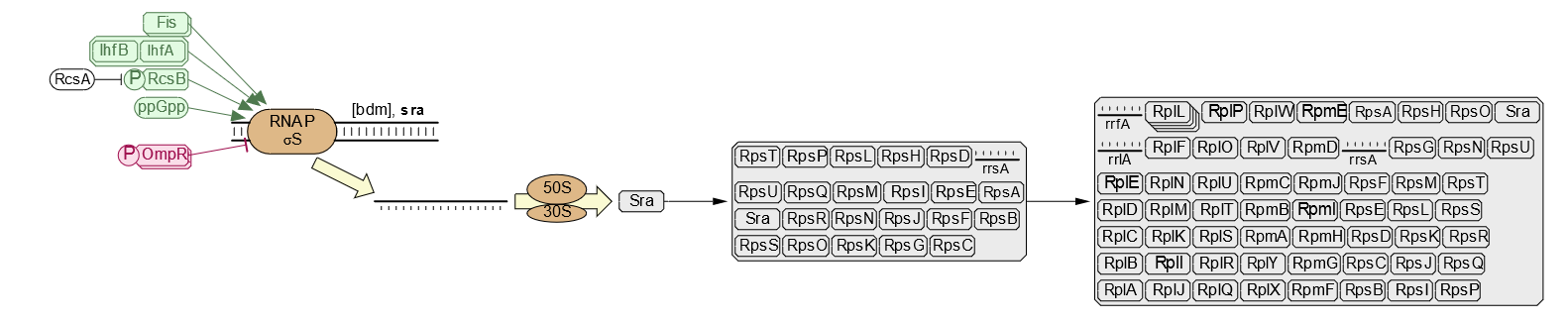 |
| **ugpB** | 0,7 | 0,7 | 0,23 | 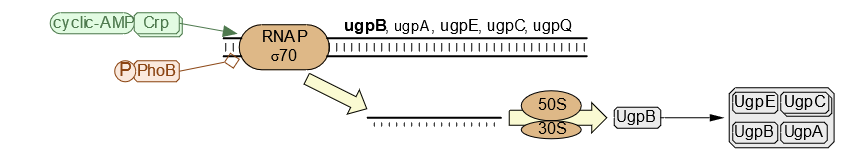 |
| **ybeD** | 0,9 | 0,6 | 3,5 | 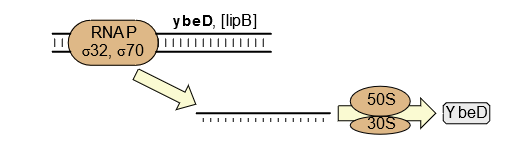 |

**Supplementary Figure 5.** Comparison of proteome and transcriptome changes caused by *rsmF* gene inactivation. Relative amount of mRNAs was estimated by RT qPCR. Relative amount of proteins measured by shot gun proteome was taken from Table 2. Regulation schemes were downloaded from EcoCyc site (Keseler, 2013).

**Supplementary references**

Keseler, I., Mackie, A., Peralta-Gil, M., Santos-Zavaleta, A., Gama-Castro, S., Bonavides-Martínez, C. et al. (2013). EcoCyc: fusing model organism databases with systems biology. *Nucleic Acids Res.* 41, D605-612.
